# Baseline ALBI Grade Predicts Benefits After Splenectomy for Cirrhotic Patients with Hypersplenism

**DOI:** 10.1007/s11605-023-05610-2

**Published:** 2023-02-09

**Authors:** Qi Zhu, Desheng Chen, Yichao Lou, Xueqian Xie, Yi Wu, Zhaowen Wang, Hongcheng Sun

**Affiliations:** 1 Department of General Surgery, Shanghai General Hospital, Shanghai Jiao Tong University School of Medicine, Shanghai, 200080 China; 2 Department of Radiology, Shanghai General Hospital, Shanghai Jiao Tong University School of Medicine, Shanghai, 200080 China

**Keywords:** Cirrhosis, Hypersplenism, Splenectomy, Cirrhosis regression, Health-related quality of life

## Abstract

**Purpose:**

Splenectomy is an effective treatment for correcting cytopenia caused by hypersplenism secondary to cirrhosis. However, other potential benefits have not been well characterized. In this study, we investigated the value of splenectomy as it relates to improvement in hepatic function, liver regeneration, and health-related quality of life, and their association with baseline characteristics to clarify which patients may benefit the most from splenectomy.

**Methods:**

Patients with hypersplenism secondary to cirrhosis treated by splenectomy were retrospectively reviewed. Hepatic function was reflected by hematologic indices and albumin-bilirubin score. Liver volume was measured by imaging software, and quality-of-life was assessed by a 36-question short-form questionnaire. The changes in these three aspects after splenectomy were evaluated in the whole cohort and compared between subgroups.

**Results:**

The hepatic function of the patients significantly improved after splenectomy, and this was reflected by elevated serum albumin, shortened prothrombin time, and decreased albumin-bilirubin score. Patients with baseline albumin-bilirubin grade 2 or 3 and age < 56 years showed significantly decreased albumin-bilirubin score after splenectomy, whereas other subgroups did not. Moreover, liver volume increased remarkably after splenectomy in patients with baseline albumin-bilirubin grade 1, but not in those with grade 2 or 3. Significant improvement in quality-of-life occurred in the entire cohort after splenectomy, but more profound improvement was found in patients with albumin-bilirubin grade 2 or 3.

**Conclusions:**

Splenectomy improves hepatic function, increases liver volume, and also improves quality-of-life in different subsets of patients with cirrhosis and hypersplenism. Baseline characteristics, such as albumin-bilirubin grade and age, are helpful in estimating the potential benefits of splenectomy for patients before surgery.

**Supplementary Information:**

The online version contains supplementary material available at 10.1007/s11605-023-05610-2.

## Introduction

Chronic liver injury can lead to persistent inflammation and liver cirrhosis (LC), which remains a common problem worldwide.^1^ LC was previously regarded as a final stage of advanced liver disease that was irreversible. Nevertheless, current clinical evidence suggests that liver fibrosis or even cirrhosis can be reversed after profound suppression of the underlying cause of the disease. For example, long-term antiviral therapy for chronic hepatitis B or C may prevent hepatic decompensation and elicit reversal of cirrhosis, thereby leading to improvement in patient prognosis.^2,3^ Furthermore, stable improvement of liver function (within Child–Pugh A and/or MELD score < 10) has been achieved after antiviral treatment in more than half of patients with hepatitis-B-related decompensated cirrhosis.^4^ However, most patients with advanced cirrhosis and hypersplenism who enter surgical departments have already had a long history of medication against etiological factors, and thus other effective therapies aimed at reversing the functional and structural deterioration of cirrhosis are still urgently needed.

LC gives rise to portal hypertension and hypersplenism, and hypersplenism in turn promotes hepatic inflammation and progression of LC.^5^ Splenectomy is therefore effective for correcting cytopenia and relieving portal hypertension in patients with hypersplenism secondary to LC.^6^ Moreover, splenectomy has been demonstrated to improve liver function in a subset of patients with preoperative Child–Pugh (C-P) grade B.^7,8^ In addition, histological examinations have revealed the regeneration of hepatic parenchyma and regression of fibrotic septa after splenectomy in 4 out of 7 patients with LC and hypersplenism.^9^ Increase in liver volume, a sign of liver regeneration and cirrhosis regression, has also been detected by imaging in cirrhotic patients with better indocyanine green (ICG) clearance and higher albumin level after splenectomy.^10^ Therefore, splenectomy may exert different feedback influence on hepatic function and LC for patients with different baseline liver function. Considering that postoperative complications are not rare and can be serious in the context of LC,^11^ it is of great importance to determine which patients are most likely to benefit from splenectomy before surgery.

Health-related quality of life (HRQL) is a subjective multidimensional perception that is used to assess patient’s physical, social, and mental well-being. Due to the symptoms and complications caused by liver dysfunction, HRQL is variably impaired in cirrhotic patients and is negatively correlated with the severity of underlying liver disease.^12,13^ Because relief of symptoms contributes to HRQL improvement after effective treatment, HRQL assessment has been utilized to evaluate therapeutic intervention for cirrhotic patients.^14,15^ It may be possible that the amelioration of liver dysfunction may relieve symptoms, thus improving the HRQL of cirrhotic patients after splenectomy. However, the invasive procedure itself as well as surgical complications may compromise HRQL, so the final impact of splenectomy on the HRQL of cirrhotic patients with hypersplenism remains unclear.

Albumin-bilirubin (ALBI) grade is a liver function assessment method that has demonstrated more objective and discriminatory power than the conventional C-P grade in cirrhotic patients.^16^ In particular, C-P grade cannot offer a wide degree of discrimination among cirrhotic patients who have undergone splenectomy, since the majority of them fall into the C-P A grade category.^8–10^ However, ALBI grade can divide cirrhotic patients with C-P grade A into more refined subgroups with clearly different prognoses.^16^ Thus, using the ALBI grade rather than the C-P grade in assessing the changes in liver function after splenectomy should be more sensitive and hence more accurate. In this study, ALBI grade and other baseline parameters were included to identify patients who are the most likely to benefit from splenectomy regarding the improvement in hepatic function, liver regeneration, and HRQL.

## Materials and Methods

### Study Cohort

We identified 1232 consecutive patients who were diagnosed with LC in the electronic record system of our hospital between December 2016 and November 2020. Among them, 367 patients were diagnosed with LC and hypersplenism. Splenectomy was generally indicated for the patients if they met the following conditions: (i) platelet count < 30–50 × 10^9^/L, leukocyte count < 2–3 × 10^9^/L, or hemoglobin < 70–80 g/L; and (ii) the presence of esophagogastric varices detected by endoscopy or enhanced CT/MRI with or without history of variceal bleeding. Splenectomy was not considered if the patients had the following contraindications: (i) uncontrolled ascites, (ii) encephalopathy, (iii) Child–Pugh C liver function, (iv) untreatable liver cancer. A total of 158 patients were eligible for splenectomy, and among them 116 patients underwent splenectomy with or without esophagogastric devascularization. These 116 patients served as the study cohort.

To avoid the interference of confounding factors, we further excluded the patients with the following conditions: (i) concomitant liver cancer or other malignancy before splenectomy; (ii) liver transplantation after splenectomy; (iii) emergency operation due to acute upper gastrointestinal hemorrhage; (iv) splenectomy combined with partial hepatectomy; (v) death due to severe postoperative complications; (vi) incomplete clinical data; and (vii) follow-up time less than 6 months. Patients with follow-up time less than 6 months were excluded for the following reasons: (i) liver function gradually improving and reaching a relatively stable state within half a year after surgery; (ii) routine oral anticoagulant treatment for preventive or therapeutic purpose within half a year after surgery (as this may interfere the accurate assessment of prothrombin time (PT)); and (iii) the potential interference of surgical complications in assessment of HRQL after surgery. This study was approved by Medical Ethics Committee of Shanghai General Hospital, Shanghai Jiaotong University School of Medicine, Shanghai, China and was registered at http://www.chictr.org (ChiCTR2100046421). Informed consent was obtained from all patients.

### Data Collection and Definitions

Demographic, clinical, and pathological data were extracted from the electronic record system of our hospital, and the diagnosis of LC was based on medical history, clinical manifestation, and laboratory, radiological, endoscopic, and histological characteristics. The diagnostic criteria for hypersplenism were as follows: (i) morphological splenomegaly; (ii) hemocytopenia (white cells, platelets, or red cells dropping below the lower limit of normal levels); and (iii) bone marrow puncture showing hematopoietic hyperplasia (used with discretion). The values of preoperative (baseline) and postoperative parameters were extracted or calculated from the results of laboratory test and imaging examinations prior to surgery and longer than 6 months after surgery, respectively. The median values of the postoperative parameters were used to compare to their corresponding baseline values if the patients had multiple follow-up data points. Liver function was conventionally evaluated by the C-P grade before and after surgery. The ALBI grade was retrospectively assessed according to the ALBI score, which was calculated from the following formula: ${\mathrm{log}}_{10}\mathrm{bilirubin }(\mathrm{\mu mol}/\mathrm{L})\times 0.66+\mathrm{albumin }(\mathrm{g}/\mathrm{L})\times (-0.085)$, and the ALBI grade was categorized into grade 1 (score ≤  − 2.60), grade 2 (score >  − 2.60 to ≤  − 1.39), or grade 3 (score >  − 1.39).^16^ Three-dimensional (3D) liver imaging was constructed, and liver volume was calculated using the Hepatic VCAR software package (GE HealthCare), which allows for the analysis and visualization of liver computed tomography (CT) data derived from DICOM 3.0-compliant CT scans. The diameter of the portal vein was measured by contrast-enhanced CT or magnetic resonance imaging (MRI) at the cross-sectional layer close to its bifurcation.

HRQL was assessed using a 36-question short-form health survey questionnaire (SF-36) that included items in the following eight dimensions: physical-functioning (PF), role-physical (RP), bodily pain (BP), general health (GH), vitality (VT), social functioning (SF), role-emotional (RE), and mental health (MH).^17^ SF-36 was routinely administrated to the patients before splenectomy (baseline HRQL) and after half a year after surgery (postoperative HRQL). The scores for each dimension can range from 0 (worst possible health state) to 100 (best possible health state).

### Perioperative Management and Follow-Up

Preoperative evaluation included the following aspects: (i) general condition, such as nutritional status and organ function; (ii) severity of primary liver disease, such as liver function, cirrhosis, and portal hypertension; (iii) possible concomitant diseases, such as liver cancer and portal vein system thrombosis (PVST); and (iv) potential surgical contraindications. Prophylactic vaccination against bacteria was not administrated for cirrhotic patients with hypersplenism before undergoing splenectomy. Splenectomy was performed to solve severe hematological disorder and relieve portal hypertension and was performed by experienced surgeons from our department under general anesthesia. Patients with a history of variceal bleeding or the presence of severe varices detected by endoscopy or enhanced CT or MRI underwent additional esophagogastric devascularization. Accessory spleen was removed together with the spleen if it had been identified prior to or during the operation. Prophylactic anticoagulation therapy was routinely started by subcutaneous administration of low-molecular-weight heparin (LMWH) 48 h after operation to prevent postoperative PVST, unless active intraperitoneal bleeding were present. Before discharge, the LMWH was replaced by oral anticoagulant. The patients were followed up with at an outpatient clinic, and monitored with blood tests, ultrasonography and/or contrast-enhanced CT/MRI every 1–3 months for the first half year after surgery, every 3–6 months for the second half year, and every 6 months thereafter.

### Statistical Analysis

Quantitative variables were expressed as median and interquartile range (IQR), and qualitative variables were expressed as frequencies and percentages. Paired quantitative and qualitative variables were compared by Wilcoxon matched-pairs signed-rank test and McNemar’s test, respectively. Univariate and multivariate logistic regression analysis was performed to identify the baseline parameters associated with improvements in liver function (decreases in ALBI score) and liver regeneration (increases in liver volume) after splenectomy. The variance inflation factor (VIF) was calculated to judge multicollinearity. Parameters with *p* < 0.1 in univariate analysis and VIF value < 5 were selected for multivariate analysis. Additionally, the receiver operating characteristic (ROC) curve was used to assess the optimal cut-off value for dichotomizing quantitative variables, and the internal consistency of the variables in the SF-36 was assessed using Cronbach’s *α* coefficient. This consistency and was deemed as adequate if the values were more than 0.7. We chose *p* < 0.05 to indicate a statistically significant test result for each of the above tests, and all statistical analysis was performed using SPSS version 24.0.0.0 (IBM, Somers, NY, USA).

## Results

### Patients’ Baseline Characteristics

After executing the exclusion criteria, a total of 71 patients were finally enrolled in this study (Supplementary Fig. 1). The median follow-up time was 18.8 (IQR 10.1–38.4) months, the cohort included 43 (60.6%) men and 28 (39.4%) women, and the median age of the patients was 51 (IQR 44–58) years. The etiologies underlying cirrhosis were hepatitis B virus (HBV) (*n* = 43, 60.6%), *Schistosomiasis japonica* (*n* = 11, 15.5%), HCV (*n* = 3, 4.2%), and others (*n* = 14, 19.7%). According to C-P grade, 54 patients (76.1%) were class A, and 17 (23.9%) were class B. For ALBI score, 20 (28.2%) patients were classified as ALBI grade 1, 43 (60.6%) as grade 2, and 8 (11.2%) as grade 3. For statistical convenience, patients with ALBI grade 3 were integrated into those with ALBI grade 2. The detailed baseline characteristics of the study cohort are summarized in Table 1.

**Table 1:** Baseline characteristics of the study cohort

Characteristic	Value
Age, year	51 (44–58)
Gender	
Male/female	42 (59.2)/29 (40.8)
Etiology	
Hepatitis B virus/*Schistosomiasis japonica*/hepatitis C virus/others	43 (60.6) / 11 (15.5) / 3(4.2%)/14 (19.7%)
History of variceal hemorrhage	
With/without	29 (40.8) / 42 (59.2)
Concomitant PVST	
With/without	7 (9.9) / 64 (90.1)
Hemoglobin, g/L	103 (83-128)
Leukocyte count, × 10^9^/L	2.62 (1.85-3.44)
Platelet count, × 10^9^/L	45.0 (35.0-60.5)
AST, U/L	33.00 (24.63-46.23)
ALT, U/L	28.0 (17.9-42.1)
Serum albumin, g/L	35.6 (32.3-40.6)
Serum total bilirubin, μmol/L	19.7 (14.9-28.6)
Serum creatinine, μmol/L	61.8 (52.6-75.9)
PT, s	14.5 (13.1-15.5)
INR	1.2 (1.1-1.3)
ALBI grade	
1/2/3	20 (28.2) / 43 (60.6) / 8 (11.2)
Child–Pugh grade	
A/B	54 (76.1) / 17 (23.9)
Esophagogastric devascularization	
With/without	44 (62.0) / 27 (38.0)
Operation method	
Laparoscopy/laparotomy	24 (33.8) / 47 (66.2)
Liver volume, cm^3^	914 (825.0-1048.5)
Spleen volume, cm^3^	776 (547.0-1005.0)
Liver/spleen volume ratio	1.23 (0.91-1.76)

Values are presented as median (IQR) for quantitative variables and *n* (%) for categorical variables

Abbreviations: *PVST*, portal venous system thrombosis; *AST*, aspartate aminotransferase; *ALT*, alanine aminotransferase; *PT*, prothrombin time; *INR*, international normalized ratio; *ALBI*, albumin-bilirubin

### Improvement of Liver Function After Splenectomy and its Association with Baseline Characteristics

**Fig. 1:**
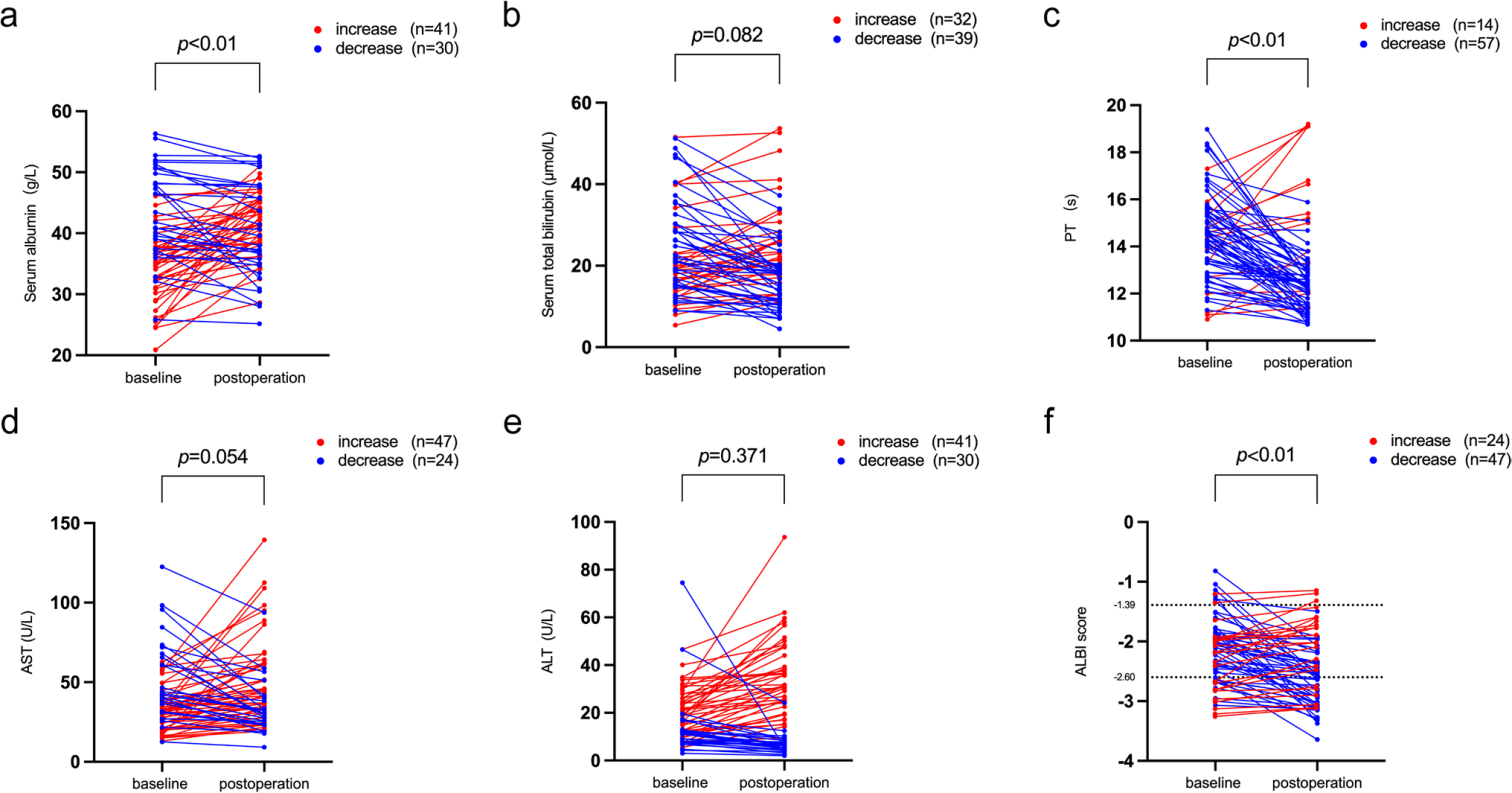
Change in hematological indices related to liver function in cirrhotic patients with hypersplenism after splenectomy (*n* = 71). **a** Serum albumin, **b** serum total bilirubin, **c** PT, **d** AST, **e** ALT, and **f** ALBI score. Differences between paired baseline and postoperative values were examined by Wilcoxon matched-pairs signed rank test. Abbreviations: PT, prothrombin time; AST, aspartate transaminase; ALT, alanine transaminase; ALBI, albumin-bilirubin

We observed drastic elevation of hemoglobin, leukocytes, and platelet counts, as well as significant decreases in portal vein diameter after splenectomy (Supplementary Fig. 2), suggesting that splenectomy was useful for treating cytopenia and portal hypertension. In addition, we found significant improvement of hepatic function for the entire cohort after splenectomy, which was reflected by elevated serum albumin, shortened PT, and decreased ALBI score (Fig. 1). Nevertheless, the change in C-P grade between baseline and post-operation was not significant (Supplementary table 1). Forty-seven (66.2%) patients displayed decreased ALBI score after surgery, and 24 (33.8%) showed an increase (Fig. 1f), indicating a different effect from splenectomy on hepatic function on different individuals.

In order to identify patients who were the most likely to benefit from splenectomy, logistic regression was performed to analyze baseline characteristics in association with the decrease in ALBI score after surgery. As a result, age (odds ratio (OR) 0.943, 95% confidence interval (CI) 0.897–0.992) and ALBI grade (OR 0.237, 95% CI 0.070–0.804) were found to be independently associated with the decrease in ALBI score after splenectomy (Table 2).

**Table 2:** Logistic regression analysis of baseline characteristics in association with the decrease of ALBI score after splenectomy (*n* = 71)

Baseline characteristics	Univariate		Multivariate	
	OR (95% CI)	*p* value	OR (95% CI)	*p* value
Age, years	0.960 (0.918–1.004)	0.077	0.943 (0.897–0.992)	0.023
Gender (male vs. female)	1.493 (0.550–4.056)	0.432		
Etiology (*Schistosomiasis japonica* vs. others)	0.357 (0.096–1.322)	0.123		
ALBI grade (1 vs. 2 or 3)	0.378 (0.130–1.104)	0.075	0.237 (0.070–0.804)	0.021
Child–Pugh grade (A vs. B)	0.523 (0.150–1.825)	0.309		
Platelet count, *${10}^{9}/\mathrm{L}$	0.994 (0.973–1.015)	0.558		
Leukocyte count, *${10}^{9}/\mathrm{L}$	1.045 (0.847–1.291)	0.679		
Hemoglobin, g/L	0.998 (0.980–1.016)	0.845		
AST, U/L	1.007 (0.983–1.032)	0.550		
ALT, U/L	1.010 (0.983–1.038)	0.458		
Serum albumin, g/L	0.935 (0.858–1.020)	0.129		
Serum total bilirubin, μmol/L	1.001 (0.958–1.047)	0.953		
Serum creatinine, μmol/L	0.999 (0.973–1.025)	0.911		
PT, s	1.072 (0.815–1.411)	0.618		
INR	2.134 (0.083–55.010)	0.647		
Operation method (laparoscopy vs. laparotomy)	0.594 (0.213–1.654)	0.319		
Concomitant PVST (with vs. without)	1.310 (0.235–7.306)	0.758		
Esophagogastric devascularization (with vs. without)	0.967 (0.351–2.666)	0.948		
Liver volume, cm^3^	1.001 (0.998–1.003)	0.504		
Spleen volume, cm^3^	1.000 (0.999–1.003)	0.858		
Liver/spleen volume ratio	1.434 (0.570–3.607)	0.444		

Abbreviations: *ALBI*, albumin-bilirubin; *AST*, aspartate aminotransferase; *ALT*, alanine aminotransferase; *PT*, prothrombin time; *INR*, international normalized ratio; *PVST*, portal venous system thrombosis; *CI*, confidence interval; *OR*, odds ratio

### Subgroup Analysis of the Change in ALBI Score After Splenectomy

**Fig. 2:**
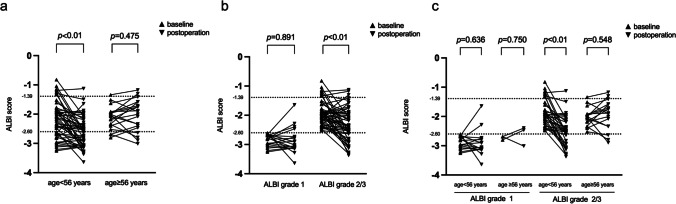
Subgroup analyses of the change in ALBI score in cirrhotic patients with hypersplenism after splenectomy (*n* = 71). Change in ALBI score after splenectomy subgrouped by age (< 56 and ≥ 56) (**a**), baseline ALBI grade (1 and 2 or 3) (**b**), and in combination (**c**). Differences between paired baseline and postoperative ALBI score were examined by Wilcoxon matched-pairs signed rank test. Abbreviations: ALBI, albumin-bilirubin

ROC analysis revealed that 55.5 years was the best cut-off value to dichotomize age for subgroup analysis. As shown in Fig. 2a, patients with age < 56 years experienced a significant decrease in ALBI score after splenectomy, whereas those with age ≥ 56 years did not. As shown in Fig. 2b, patients with baseline ALBI grade 2 or 3 had significant decreases in ALBI score after surgery, whereas those with baseline ALBI grade 1 did not. After combining baseline age and ALBI grade, the subgroup with age < 56 years and ALBI grade 2 or 3 showed the most significant decrease in ALBI score after splenectomy, and the subgroup with age ≥ 56 years and ALBI grade 2 or 3 as well as the subgroup with ALBI grade 1 (regardless of age) did not (Fig. 2c).

### The Influence of Splenectomy on Liver Volume and its Association with Baseline ALBI Grade

**Fig. 3:**
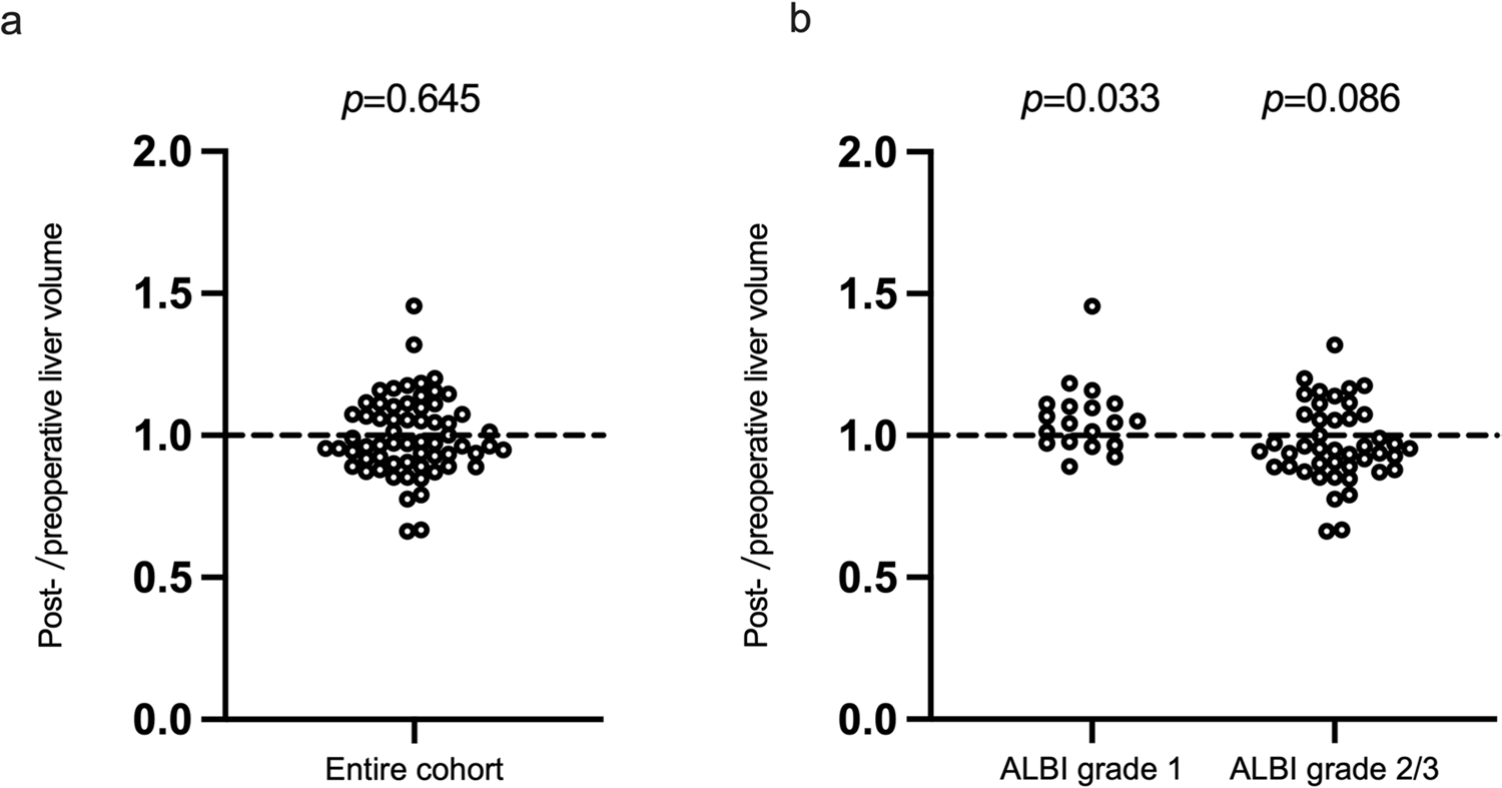
Change in liver volume in cirrhotic patients with hypersplenism after splenectomy (*n* = 63). **a** Post-/preoperative liver volume ratios for the entire cohort. **b** Subgroup analysis of post-/preoperative liver volume ratios based on baseline ALBI grade. Each dot represents the volume ratio of one patient, ratio > 1 (above the datum line) means increased liver volume after splenectomy, and ratio < 1 (below the datum line) indicates decreased liver volume after splenectomy. Differences between paired liver volumes were examined by Wilcoxon matched-pairs signed rank test

We excluded 8 patients from our liver-volume-related analysis due to missing CT scan data, leaving 63 patients who were eligible for the following study. As demonstrated in Fig. 3a, 28 (44.4%) patients displayed increases in liver volume after splenectomy, although the entire cohort failed to show a statistically significant change in liver volume after surgery (*p* = 0.645). Logistic regression analysis revealed that ALBI grade (OR 4.098, 95% CI 1.146–14.657) was the only independent baseline characteristic associated with liver volume enlargement after splenectomy (Table 3). Furthermore, subgroup analysis demonstrated significant increase in liver volume after splenectomy in patients with baseline ALBI grade 1, but a tendency of decrease in liver volume in those with baseline ALBI grade 2 or 3 (Fig. 3b).

**Table 3:** Logistic regression analysis of baseline characteristics in association with enlargement of liver volume after splenectomy (*n* = 63)

Baseline characteristics	Univariate		Multivariate	
	OR (95% CI)	*p* value	OR (95% CI)	*p* value
Age, years	0.952 (0.907–1.000)	0.048	0.983 (0.930–1.039)	0.540
Gender (male vs. female)	1.292 (0.470–3.548)	0.619		
Etiology (*Schistosomiasis japonica* vs. others)	0.206 (0.040–1.046)	0.057	0.326 (0.056–1.883)	0.210
ALBI grade (1 vs. 2 or 3)	5.413 (1.636–17.907)	0.006	4.098 (1.146–14.657)	0.030
Child–Pugh grade (A vs. B)	1.179 (0.356–3.908)	0.787		
Platelet count, *${10}^{9}/\mathrm{L}$	1.000 (0.979–1.021)	0.993		
Leukocyte count, *${10}^{9}/\mathrm{L}$	1.016 (0.839–1.229)	0.874		
Hemoglobin, g/L	1.000 (0.982–1.018)	0.977		
AST, U/L	0.984 (0.961–1.009)	0.210		
ALT, U/L	0.985 (0.956–1.015)	0.322		
Serum albumin, g/L	1.076 (0.985–1.175)	0.104		
Serum total bilirubin, μmol/L	1.003 (0.960–1.049)	0.879		
Serum creatinine, μmol/L	1.016 (0.988–1.044)	0.265		
PT, s	1.136 (0.865–1.491)	0.359		
INR	3.645 (0.141–94.407)	0.436		
Operation method (laparoscopy vs. laparotomy)	0.544 (0.188–1.575)	0.262		
Concomitant PVST (with vs. without)	3.333 (0.595–18.637)	0.171		
Esophagogastric devascularization (with vs. without)	1.754 (0.621–4.953)	0.288		
Liver volume, cm^3^	1.000 (0.999–1.002)	0.795		
Spleen volume, cm^3^	1.000 (0.999–1.002)	0.522		
Liver/spleen volume ratio	0.497 (0.190–1.296)	0.153		

Abbreviations: *ALBI*, albumin-bilirubin; *AST*, aspartate aminotransferase; *ALT*, alanine aminotransferase; *PT*, prothrombin time; *INR*, international normalized ratio; *PVST*, portal venous system thrombosis; *CI*, confidence interval; *OR*, odds ratio

### Improvement in HRQL After Splenectomy and its Association with Baseline ALBI Grade

**Fig. 4:**
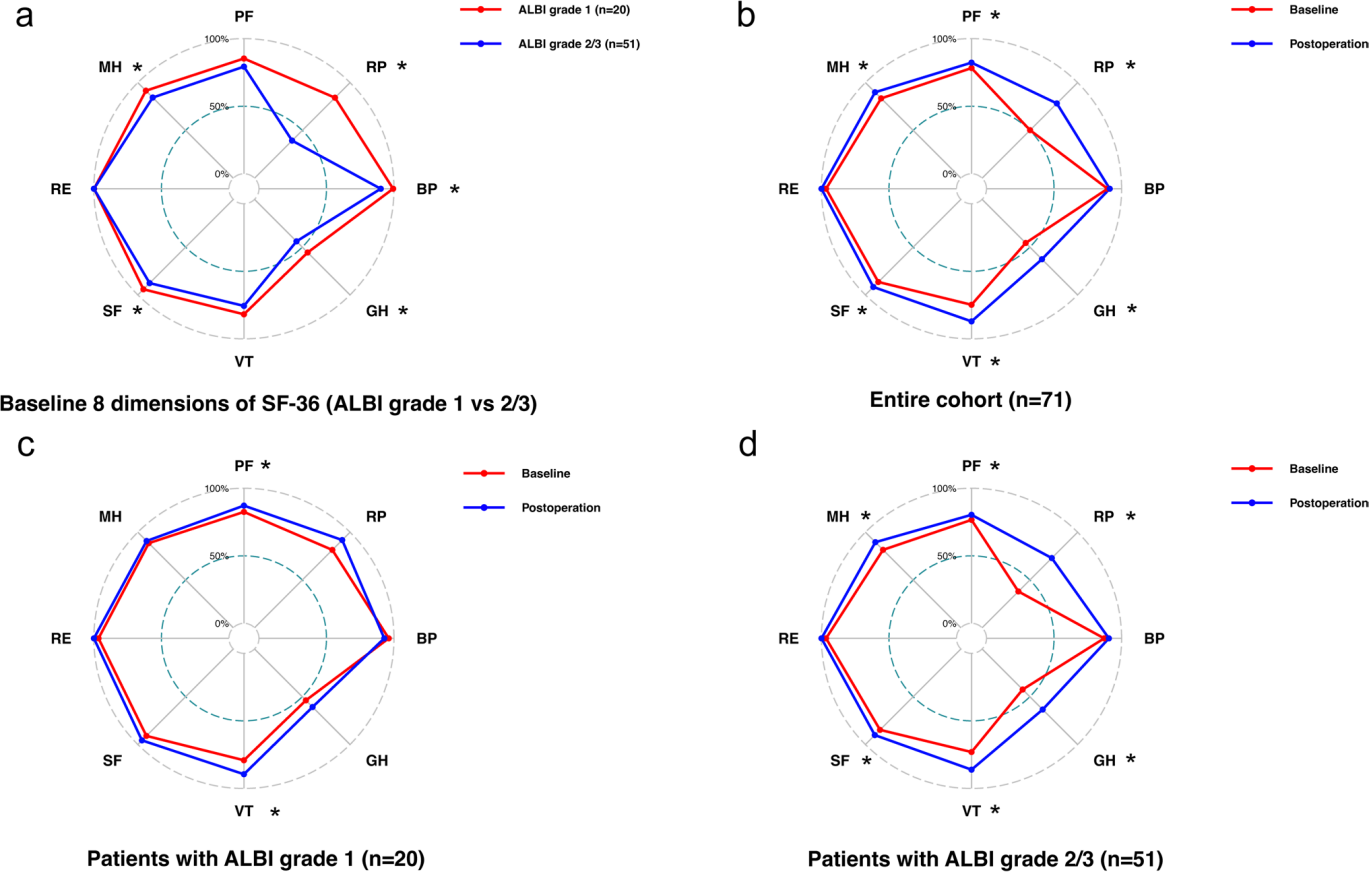
Different baseline health-related quality of life (HRQL) and change in HRQL after splenectomy in cirrhotic patients with hypersplenism stratified by baseline ALBI grade. **a** Radar plot displaying different baseline HRQL in patients with baseline ALBI grade 1 (red line) and grade 2 or 3 (blue line). Radar plot demonstrating the difference between baseline HRQL (red line) and postoperative HRQL (blue line) in the entire cohort (*n* = 71) (**b**), in patients with baseline ALBI grade 1 (*n* = 20) (**c**), and in those with baseline ALBI grade 2 or 3 (*n* = 51) (**d**). HRQL was assessed by 8 dimensions (BP, GH, MH, PF, RE, RP, VT, and SF) of the SF-36 questionnaire. Differences between paired scores in each dimension were examined by Wilcoxon matched-pairs signed rank test. *, *p* < 0.05. Abbreviations: SF-36, 36-question short-form health survey questionnaire; BP, bodily pain; GH, general health; MH, mental health; PF, physical functioning; RE, role-emotional; RP, role-physical; VT, vitality; SF, social functioning

The reliability of the SF-36 in all of the 8 dimensions of HRQL was evaluated by Cronbach’s coefficient *α* as described above, and they were all found to be reliable (data not shown). Patients with baseline ALBI grade 1 showed better performance in the RP, BP, GH, SF, and MH dimensions in comparison to patients with baseline ALBI grade 2 or 3 (Fig. 4a). The entire cohort demonstrated significant improvement in the PF, RP, GH, VT, SF, and MH dimensions after splenectomy (Fig. 4b). In patients with baseline ALBI grade 1, significant improvement was only found in the PF and VT dimensions after splenectomy (Fig. 4c). However, in patients with baseline ALBI grade 2 or 3, profound improvements were shown in the dimensions of PF, RP, GH, VT, SF, and MH after surgery (Fig. 4d).

### Surgical Complications

Among the 116 patients who underwent splenectomy, 37 (31.9%) suffered at least one surgical complication. As shown in Supplementary Table 2, severe complications (Clavien-Dindo grade ≥ III) occurred in 9 (7.8%) patients. Among them, 8 (6.9%) patients required surgical intervention for abdominal hemorrhage (6 patients) and incision hernia (2 patients), and 1 (0.9%) patient died after emergency splenectomy with esophagogastric devascularization due to uncontrolled postoperative bleeding. PVST was detected in 23 (19.8%) patients, 22 of them were asymptomatic, and 1 patient manifested decompensated liver function but finally survived after anticoagulation therapy.

## Discussion

Hypersplenism and portal hypertension are common clinical manifestations of LC and are indicative of severe liver disease and an increased risk of decompensation and complications.^18^ Splenectomy is an effective treatment option to increase platelet and white blood cell counts and alleviate portal hypertension for cirrhotic patients and hypersplenism.^6^ However, some scholars have proposed pharmacological treatment of hypersplenism instead of surgery for the following reasons. First, pharmacological agents are also effective in correcting cytopenia. Second, the benefits of splenectomy other than raising the platelet and leucocyte counts are still controversial.^19^ This proposal has been challenged by accumulating laboratory and clinical evidence that has demonstrated improvement of hepatic function after splenectomy in cirrhotic patients with hypersplenism.^5,7–10^ In this study, we observed significantly elevated serum albumin, shortened PT, and decreased ALBI score in addition to raised hemoglobin, leukocytes, and platelet counts, and reduced portal vein diameter after surgery, indicating that improvement in liver function was an additional benefit of splenectomy for cirrhotic patients. Nevertheless, when assessed by C-P grade, the change in hepatic function after splenectomy did not reach statistical significance (*p* = 0.263). Thus, C-P grade may not be sensitive enough to evaluate the change in liver function due to its low discriminative power in patients with compensated liver function, such as those who accounted for the majority of the present cohort, whereas ALBI score is a continuous, objective, and discriminatory index of liver function, and ALBI grade highlights distinct prognostic subgroups within C-P grade A.^16,20^ Therefore, ALBI score/grade should be more applicable than C-P classification model in assessing patients’ hepatic function after splenectomy.

Although improvement in hepatic function after splenectomy has been previously reported in a number of patients with LC,^5,7–10^ it still remains unclear who may benefit from it. Yamamoto et al. demonstrated marked change of C-P grade (from B to A) in 14 of 18 cirrhotic patients with hypersplenism after splenectomy.^8^ Likewise, another study, aiming to investigate the benefits of splenectomy and curative treatments for patients with hepatocellular carcinoma and portal hypertension, showed that liver function was converted to C-P grade A in 98 of 101 patients who had preoperative C-P grade B after surgery.^7^ These findings indicated that patients with decompensated liver function (C-P grade B) were likely to benefit from splenectomy. Nevertheless, no improvement in liver function could be measured in patients with C-P grade A. In the present study, logistic regression analysis of a panel of baseline characteristics revealed the significant association of age and ALBI grade with decreased ALBI score after splenectomy. Patients with age < 56 years or those with baseline ALBI grade 2 or 3 were inclined to show decreased ALBI score after splenectomy. Liver function physiologically declines in elderly patients due to reduced number of hepatocytes, decreased liver weight, and diminished hepatic blood inflow.^21^ The negative influence of aging on the liver might be the reason why splenectomy could not elicit significant improvement of liver function in cirrhotic patients with age ≥ 56. In addition, insignificant decreases in ALBI score after splenectomy in patients with baseline ALBI grade 1 may be attributed to small values of the ALBI score before surgery, namely, the ceiling effect. After integration of age and baseline ALBI grade, only patients with both age < 56 years and ALBI grade 2 or 3 showed significant decrease in ALBI score after splenectomy, suggesting that this subgroup of cirrhotic patients tended to acquire improved liver function after splenectomy. This group should thus be considered the most appropriate candidates for the surgical treatment.

Cirrhosis is not a static stage of chronic liver disease, but a dynamic pathological process during which liver fibrosis and hepatic regeneration are transformable.^22^ We believe that the splenomegaly and hypersplenism facilitated the progression of liver fibrosis to cirrhosis, and thus splenectomy was able to initiate the reversal of liver cirrhosis. The mechanistic contributions of splenomegaly and hypersplenism to the development of liver cirrhosis have been well reviewed in the literature.^5^ Specifically, TGF-β1 and cytokines (such as IL-6) produced by splenic macrophages can affect the progression of liver fibrosis and regeneration in patients with liver cirrhosis via liver-spleen cross-talk.^23^ Moreover, hepatic stellate cells and Kupffer cells act as the effectors of collagen deposition and inflammation modulation with the aid of the pro-fibrogenic cytokine TGF-β1,^24^ and this in turn damages the liver parenchyma and vascular structures.^25^ In addition, splenic contributions to liver fibrosis may be mediated by the migration of splenic immune cells (e.g., monocytes, Treg cells) to the cirrhotic liver.^5^

It is clear that not every patient can benefit from splenectomy vis-à-vis cirrhotic reversal, however. Theoretically, the reversal of liver cirrhosis relies on three different mechanisms: degradation of the extracellular matrix (ECM), replacement of vanishing fibrotic tissue by newly formed hepatocytes, and restoration of a lobular architecture with translobular blood flow.^26^ Nonetheless, in clinical practice, it is still unknown which patients are most likely to benefit from cirrhotic reversal after splenectomy. Our results demonstrated that baseline ALBI grade was the only factor associated with enlargement of liver volume after splenectomy and that patients with baseline ALBI grade 1 showed significant increase in liver volume. These findings support the notion that baseline ALBI grade may have the power to differentiate patients who are most likely to experience cirrhotic regression after splenectomy.

The reversibility of hepatitis B virus cirrhosis after long-term administration of antiviral therapy depends on the severity of LC. That is, mild cirrhosis is more likely to be reversed than severe cirrhosis.^26^ According to the fact that smaller ALBI scores indicated milder fibrous degrees in patients with LC,^27^ we can deduce that patients with baseline ALBI grade 1 in this cohort should have had less serious cirrhosis and were therefore more likely to experience reversal of cirrhosis. However, those with ALBI grade 2 or 3 may have had relatively severe cirrhosis, and under such circumstances, extensive accumulation of collagen and elastic fibers as well as exhausted regeneration of hepatocytes may have made cirrhotic regression less likely to occur after splenectomy.

Our data also revealed that improvement of HRQL is another benefit of splenectomy in LC patients with hypersplenism. At baseline, relatively poor HRQL was shown in our patients with ALBI grade 2 or 3 in comparison to those with grade 1, which was in line with the fact that HRQL decreases with liver decompensation (C-P grade B/C) in cirrhotic patients.^28^ These findings suggest that liver function status positively correlates with the HRQL of patients with LC. Interestingly, despite experiencing surgery-related discomfort, the entire cohort still showed significant improvement in 6 out of 8 dimensions of HRQL after splenectomy. This result may be attributed to improved liver function after surgery, since restoration of hepatic anabolic metabolism and ammonia disposal could reverse the proteolytic process of skeletal muscle and alleviate encephalopathy, thereby contributing to improvement in physical and mental well-being.^29,30^ Our data also showed that patients with baseline ALBI grade 2 or 3 gained more profound improvement in HRQL than those with ALBI grade 1, which can be explained by the following reasons. First, patients with baseline ALBI grade 2 or 3 were more likely to gain significantly improved liver function after splenectomy. Second, patients with ALBI grade 2 or 3 usually had poor baseline HRQL and may have had an easier time perceiving HRQL improvement.

Although our study provided important evidence regarding how cirrhotic patients benefit from splenectomy according to their baseline characteristics, it did have some limitations. First, hepatitis B virus was the main cause of cirrhosis in this cohort, so whether our results still hold true in patients dominated by other etiologies needs further study. Second, we used change of liver volume, measured by CT scan, for indirect evaluation of liver regeneration as previously reported.^10^ Liver biopsy in cirrhotic patients with hypersplenism is extremely dangerous due to the high possibility of uncontrolled hemorrhage, because these patients always present severe thrombocytopenia, coagulopathy, and portal hypertension. That is why this ideal but infeasible diagnostic method is not routinely performed before splenectomy. In future studies, development of more reliable, noninvasive, and easily accessible parameters that can be used in place of liver biopsy is of particular importance in assessment of cirrhotic reversal after splenectomy. Third, due to the retrospective nature of this study, our conclusions need to be evaluated prospectively in larger cohorts from multiple centers.

## Conclusion

Our data showed that splenectomy not only corrected cytopenia and relieved portal hypertension but also improved hepatic function, increased liver volume, and also improved HRQL in different subsets of patients with LC and hypersplenism. Baseline characteristics, such as ALBI grade and age, were helpful in estimating the potential benefits of splenectomy for patients prior to surgery. Therefore, we suggest that clinicians conduct a careful evaluation of baseline parameters in order to deliver the best treatment for cirrhotic patients with hypersplenism.

## Supplementary Information

Below is the link to the electronic supplementary material.

Supplementary file1 (DOCX 52 KB)

Supplementary file2 (TIFF 1598 KB)

Supplementary file3 (TIFF 281 KB)

## Data Availability

These data are not publicly available due to the information of these data could compromise the privacy of participants enrolled in this research.
