# Supplementary material for: Baseline ALBI Grade Predicts Benefits After Splenectomy for Cirrhotic Patients with Hypersplenism
Source: J Gastrointest Surg. 2023 Feb 9;27(6):1130–40. doi: 10.1007/s11605-023-05610-2 (PMC10267274; doi:10.1007/s11605-023-05610-2)
Supplement: Supplementary file 1 — Supplementary file1 (DOCX 52 KB) [file 11605_2023_5610_MOESM1_ESM.docx]

**Supplementary Table 1. Change in C-P grade for cirrhotic patients with hypersplenism before and after splenectomy (n=71)**

| Baseline | Postoperation | | Total |
| --- | --- | --- | --- |
|  | C-P grade A | C-P grade B |  |
| C-P grade A  C-P grade B | 47  13 | 7  4 | 54  17 |
| Total | 60 | 11 | 71 |

*p*=0.263^a^

^a^Differences between paired Child-Pugh grades were examined by McNemar’s test.

*Abbreviations:* C-P, Child-Pugh.

**Supplementary Table 2. Surgical complications after splenectomy graded by the Clavien-Dindo classification system (n=116).**

| Surgical complication | Clavien-Dindo grade | n (%) |  |
| --- | --- | --- | --- |
|  |  |  |  |
| Pancreatic fistula | I | 3 (2.6) | |
| Transient elevation of serum creatinine | I | 3 (2.6) | |
| Wound infection | I | 5 (4.3) | |
| Fever (>38.5°C, unknown origin) | II | 7 (6.0) | |
| Pneumonia | II | 1 (0.9) | |
| Esophageal fistula | II | 1 (0.9) | |
| Gastric motility disorder | II | 3 (2.6) | |
| Central venous catheter infection | II | 3 (2.6) | |
| Portal vein system thrombosis | II | 23 (19.8) | |
| Blood transfusion | II | 4 (3.4) | |
| Incision hernia | III b | 2 (1.7) | |
| Intra-abdominal hemorrhage | III b | 6 (5.2) | |
| Death | V | 1 (0.9) | |
