# Supplementary figures and images for: Baseline ALBI Grade Predicts Benefits After Splenectomy for Cirrhotic Patients with Hypersplenism
Source: J Gastrointest Surg. 2023 Feb 9;27(6):1130–40. doi: 10.1007/s11605-023-05610-2 (PMC10267274; doi:10.1007/s11605-023-05610-2)

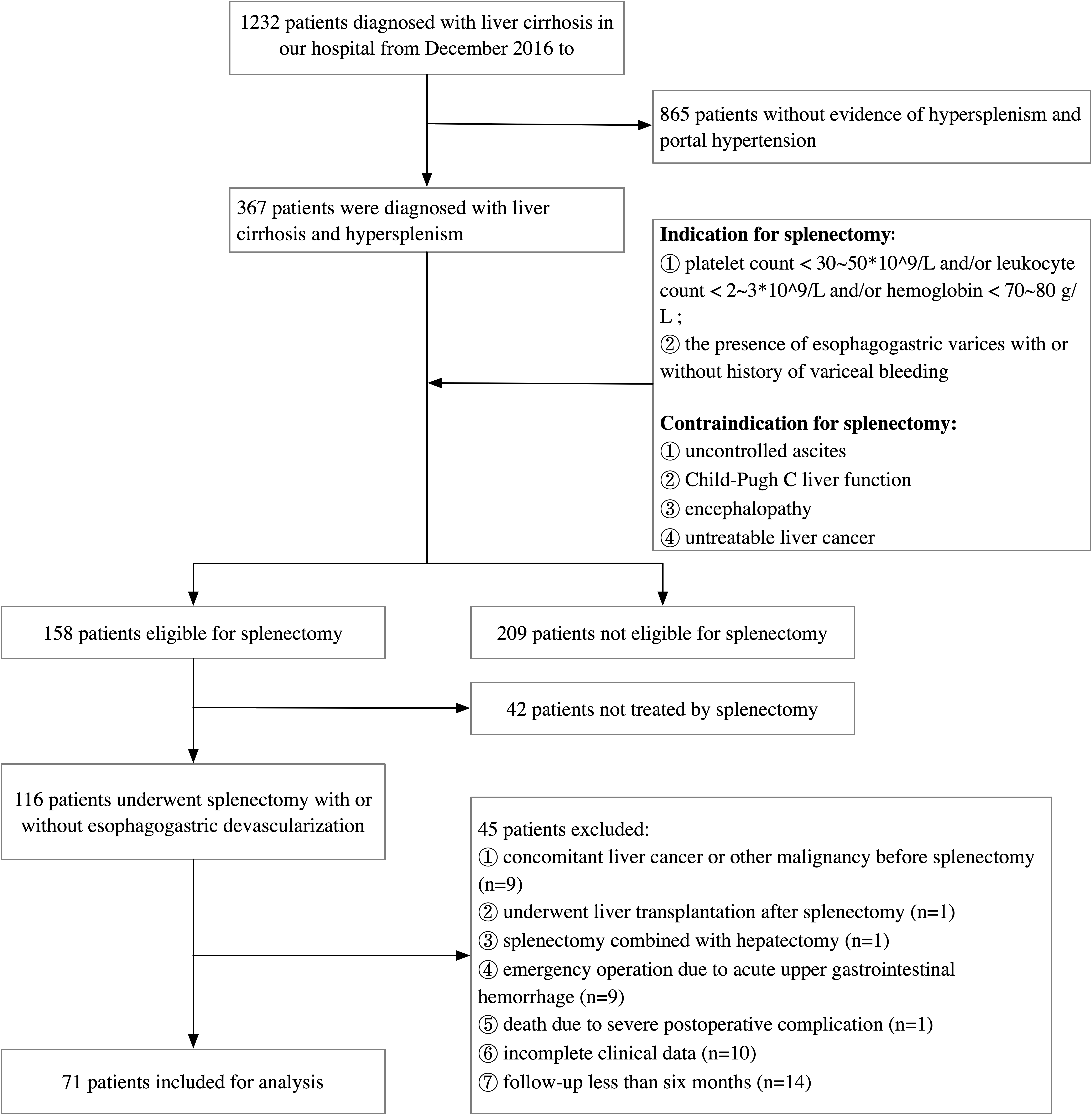

Supplement: Supplementary file 2 — Supplementary file2 (TIFF 1598 KB) [file 11605_2023_5610_MOESM2_ESM.tiff]

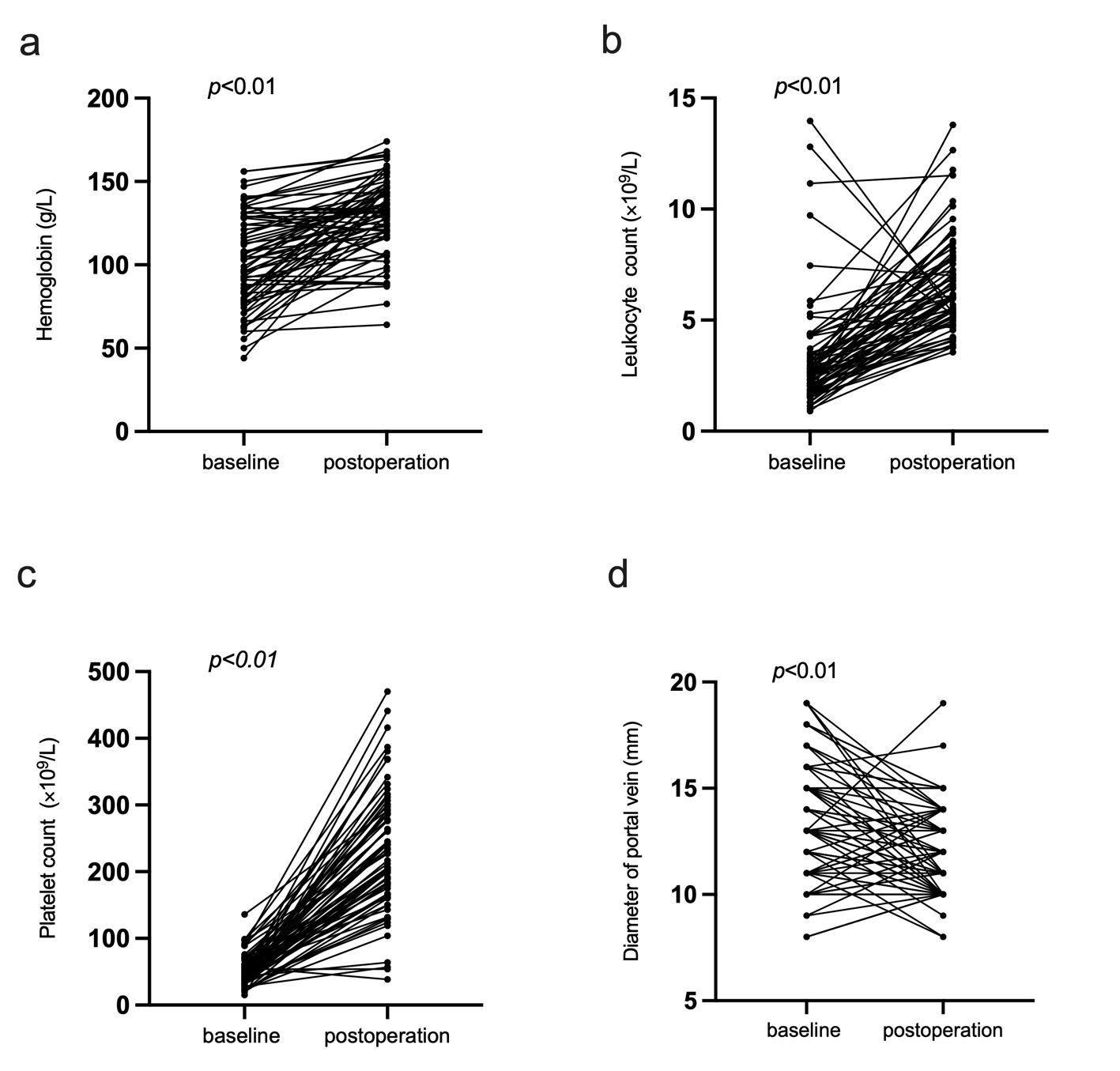

Supplement: Supplementary file 3 — Supplementary file3 (TIFF 281 KB) [file 11605_2023_5610_MOESM3_ESM.tiff]
